# Interplay between Extracellular Matrix and Neutrophils in Diseases

**DOI:** 10.1155/2021/8243378

**Published:** 2021-07-16

**Authors:** Yanyan Zhu, Yumeng Huang, Qian Ji, Shengqiao Fu, Jia Gu, Ningzheng Tai, Xu Wang

**Affiliations:** ^1^Department of Burn and Plastic Surgery, Affiliated Hospital, Jiangsu University, Zhenjiang, 212001 Jiangsu Province, China; ^2^Department of Radiation Oncology, Institute of Oncology, Affiliated Hospital of Jiangsu University, Zhenjiang, 212001 Jiangsu Province, China

## Abstract

The extracellular matrix (ECM) is a highly dynamic and complex network structure, which exists in almost all tissues and is the microenvironment that cells rely on for survival. ECM interacts with cells to regulate diverse functions, including differentiation, proliferation, and migration. Neutrophils are the most abundant immune cells in circulation and play key roles in orchestrating a complex series of events during inflammation. Neutrophils can also mediate ECM remodeling by providing specific matrix-remodeling enzymes (such as neutrophil elastase and metalloproteinases), generating neutrophil extracellular traps, and releasing exosomes. In turn, ECM can remodel the inflammatory microenvironment by regulating the function of neutrophils, which drives disease progression. Both the presence of ECM and the interplay between neutrophils and their extracellular matrices are considered an important and outstanding mechanistic aspect of inflammation. In this review, the importance of ECM will be considered, together with the discussion of recent advances in understanding the underlying mechanisms of the intricate interplay between ECM and neutrophils. A better comprehension of immune cell-matrix reciprocal dependence has exciting implications for the development of new therapeutic options for neutrophil-associated infectious and inflammatory diseases.

## 1. Introduction

Neutrophils are the most abundant leucocytes in human blood and are considered as the first line of defense against invading organisms. Neutrophils can release various cytoplasmic granules, including primary granules, secondary granules, gelatinase granules, and secretory vesicles. Primary particles are related to bactericidal function, whereas secondary and tertiary particles are related to extracellular matrix (ECM) interactions and immune modification [1]. ECM is a complex structure that is composed of the interstitial matrix and basement membrane. ECM can not only regulate the homeostasis of the tissue microenvironment through synthesis, degradation, recombination, and chemical modification, but its rich protein components and immunologically active molecules can also exert an indispensable role in immune regulation when the body responds to external stimuli [2]. Previous studies have shown that ECM has a broad impact on the basic function of immune cells, including cell survival, growth, differentiation, and migration. In addition, accumulating evidence could also suggest that various proteases released by neutrophils can participate in the continuous remodeling process of ECM and mediate immune responses. The close relationship between ECM and neutrophils plays important roles in various disease progression. Here, we review the mounting evidence of the complex interactions between ECM macromolecules and neutrophils, and highlight the potential of ECM-neutrophil interactions for the treatment of neutrophil-associated infectious and inflammatory diseases.

## 2. Extracellular Matrix

ECM is a precise and orderly network structure that is composed of macromolecules such as proteins and polysaccharides synthesized by cells and secreted into the extracellular space. ECM is composed of collagen, elastin, fibronectin, laminin, aminoglycans, and proteoglycans [3, 4]. The interstitial matrix is a loose network of collagen fibers, mainly composed of type I and III collagen, fibronectin, elastin, and various proteoglycans. The basement membrane is a dense protein network structure that is composed of type IV collagen, laminin, nestin, and heparan sulfate proteoglycan. It is located at the base of epithelial or endothelial cells, which separates cells from the surrounding matrix and acts as a barrier for material transportation [5]. ECM is a dynamic and complex tissue structure that can trigger a variety of biological activities, which are vital to the normal development of organs and the stability of the internal environment of the tissue. In recent years, much attention has been paid to the research of the ECM protein and its biological function.

### 2.1. Collagen

Collagen is the most abundant protein in mammals, accounting for about 1/3 of the total protein. It is mainly deposited in ECM which has the function of maintaining tissue shape, structure, and mechanical properties [6]. In vertebrates, 28 types of collagens have been described (I-XXVIII), which are divided into several families. Each type of collagen fiber is composed of several subtypes of collagen according to its tissue location.

Collagen plays a core and dynamic role in the formation of tissue immune microenvironment. Studies have found that tumors are associated with the synthesis, cross-linking, and deposition of fibrillar collagen (mainly type I). Fibrillar collagen gradually accumulates in the matrix to form a dense network of ECM fibers, leading to tissue sclerosis [7]. The deficiency of collagen VI may change the structure and biomechanical properties of the extracellular matrix, leading to increased apoptosis and oxidative stress, and impaired muscle regeneration [8]. Collagen IX is essential for the integrity of cartilage. Its lack of synthesis leads to defects in cartilage formation and the development of severe degenerative joint diseases resembling osteoarthritis [9]. In addition, collagen can cross-link various ECM proteins and guide the structural composition of ECM during the pathological process of pulmonary fibrosis, which affects the biomechanical properties of ECM to increase its hardness and stability [10].

### 2.2. Laminin

Laminin, as the main adhesion protein of the extracellular matrix, is the earliest component of the extracellular matrix in embryonic development, and it is also one of the main structural components of the basement membrane. They are primarily located in the basal lamina and some mesenchymal compartments.

Interplays between modular domains within the laminin molecule and other proteins enable laminins to mediate interactions between cells via cell surface receptors (such as integrins and dystroglycans) and other components of the ECM (such as nidogens, perlecans, and collagen) [11, 12]. Cells binding to laminin occur via a variety of receptors, including nonintegrins and integrins [11, 13]. The *β*1 family includes most of the laminin-binding integrins (*α*1*β*1, *α*2*β*1, *α*3*β*1, *α*6*β*1, *α*7*β*1, and *α*9*β*1) [14]. Other integrins that bind laminin include *α*v*β*3 and *α*6*β*4. Laminin has been shown to directly or indirectly control cell activity through interaction with cells, such as adhesion or metastasis, differentiation and polarization, proliferation or apoptosis, and gene expression [14]. Furthermore, as a major component of the cell basement membrane, laminin is involved in various physiological and pathological processes such as inflammation, immune response, and tumor metastasis in vivo. For example, laminin in the basement membrane of ECM can send signals to upregulate platelet endothelial cell adhesion molecule 1 on the surface of neutrophils during inflammation, and then upregulate the expression of integrin *α*6*β*1 receptor on its surface, promoting neutrophil transmembrane transport and infiltration of immune cells into damaged inflammatory tissues [15].

### 2.3. Proteoglycans

Proteoglycans are characterized as polymer complexes formed by covalent binding of glycosaminoglycans (except hyaluronan) and the core protein, which is the main component of connective tissue. Glycosaminoglycans are linear polysaccharides widely found on the cell surface and ECM, including hyaluronic acid, chondroitin sulfate, heparin, and heparan sulfate. Proteoglycan is an important multifunctional molecule, which has a wide range of functions in the organism. Many studies have shown that these proteoglycans in ECM play important roles in cell adhesion, migration, proliferation, and differentiation [16].

Hyaluronan (HA) is a nonsulphated glycosaminoglycan ubiquitous in ECM. When the tissue is damaged, proinflammatory fragments of hyaluronan are decomposed and released under the action of reactive oxygen species or hyaluronidases, which then recognize Toll-like receptors on the surface of immune cells and stimulate the release of proinflammatory cytokines, maintaining the inflammatory response [17]. HA can also covalently bind to a variety of proteins, such as CD44 and the receptor for HA-mediated motility (RHAMM), affecting the immune process of the body [18, 19]. Versican is a large extracellular matrix proteoglycan which exists in a variety of tissues. The versican core protein is composed of several different elements, leading to the binding of many proteins, including ECM proteins such as collagen, cytokines, transmembrane proteins, and cytoplasmic proteins. Due to its complex structure and widespread expression in vivo, versican has multiple functions, which include cell adhesion, proliferation, and migration [20]. In addition, heparin sulfate proteoglycan can be used as a ligand for selectins on the surface of leukocytes and directly participate in the regulation of leukocyte recruitment in the inflammatory environment. Heparan sulfate proteoglycans can also be decomposed into soluble fragments to fix chemokines on the surface of blood vessels and lymphatic endothelial cells, and guide the directional migration of leukocytes [21].

### 2.4. Biologically Active Fragments of the Extracellular Matrix

Numerous ECM-affiliated proteins and glycosaminoglycans (GAGs) are partially hydrolyzed to produce diverse bioactive fragments that are typically different from full-length molecules, collectively referred to as matrikines [22]. Under pathological conditions, matrikines transmit molecular signals to epithelial or immune cells through unique pattern recognition receptors, activate the immune system to eliminate pathogens, and promote repair of damaged tissues [23]. Additionally, matrikines have been confirmed to possess chemotactic properties that intact extracellular matrix molecules do not have [22]. For example, both the Laminin-332 fragment and Laminin gamma2 peptide digested by NE were found to be chemotactic for neutrophils [24]. Similarly, neutrophil-derived MMP-8 and MMP-9 form the extracellular matrix polypeptide of the PGP sequence by splitting collagen, which activates CXCR1/2 receptors to mediate neutrophil chemotaxis and increase vascular permeability, thereby promoting inflammatory cascades [22, 25]. Based on these findings, matrikines may be a long-standing alarm signal in host defense.

## 3. Effects of Neutrophil-Derived Proteases on ECM Regulation

### 3.1. Neutrophil Elastase

Neutrophil elastase (NE) is one of the most abundant serine proteases released by neutrophils. Under physiological conditions, neutrophils release a small amount of NE; however, when the organism is invaded and infected by external pathogens, inflammatory cytokines will stimulate neutrophils to release NE in large quantities to kill pathogenic microorganisms. Elevated levels of NE can digest not only elastin but also other extracellular matrix proteins, including collagen, laminin, and many transmembrane proteins through its powerful protease activity [26], which destroys the tight junctions between cells and induces the exudation and migration of neutrophils (Table 1).

NE can digest and degrade ECM and epithelial junctions by damaging capillary endothelial cells and alveolar epithelial cells, which plays a key role in the development of acute lung injury (ALI) [27]. Elastin is the main component of connective tissue in the lung parenchyma. Some studies suggested that plasma NE levels could be used as an indicator to predict and judge the occurrence and development of ALI [28]. Another study showed that the number of neutrophils in bronchoalveolar fluid (BALF) was significantly increased in patients with idiopathic pulmonary fibrosis (IPF). The quantitative analysis of NE-degraded elastin by EL-NE assay showed that the levels of NE-degraded elastin in the serum of patients with IPF and lung cancer were significantly higher than those in healthy controls [29, 30]. Moreover, the increase in NE activity leads to the activation of MMPs, which may enhance the degradation of ECM and cause tissue damage [31]. Proteases are known to play a vital role in gastrointestinal homeostasis and barrier function, and the imbalance of protease/antiprotease balance in the intestine can lead to gastrointestinal disease [32]. According to a published report, NE and elastin lytic activity in the intestinal mucosa of patients with inflammatory bowel disease are significantly increased, which increases the degradation of ECM and aggravates the destruction of the intestinal mucosal barrier, leading to erosion or ulcers [33].

### 3.2. Matrix Metalloproteinases

Matrix metalloproteinases (MMPs) are a class of important proteolytic enzymes, which are secreted by various cells and depend on metal ions such as calcium and zinc, and use ECM as the substrate for hydrolysis. Up to now, more than 20 members of the MMP family have been identified, which are divided into 6 subfamilies based on their structure and substrate specificity, including collagenases, gelatinases, matrix lysin, mesenchymal lysin, model MMPs, and other MMPs. MMPs are considered one of the most important proteolytic enzymes for degrading ECM which play a major role in pathophysiological processes.

MMPs are proteolytic metalloendopeptidases with the ability to alter the immune response and have been shown to be upregulated in patients with inflammation-related diseases. The key mechanism of its action is implicated in matrix destruction (Table 1). It is believed that neutrophil-derived MMP-8 and MMP-9 can degrade collagen in the ECM to produce bioactive peptides that promote neutrophil chemotaxis, and cause neutrophil chemotaxis by activating CXCR1/2 receptors, promoting the occurrence of inflammatory cascades [22, 25, 34]. Neutrophil infiltration is a characteristic of the immunopathology of tuberculosis, but the mechanism by which neutrophils cause lung tissue damage is still unclear. In tuberculosis, Ong et al. showed that the secretion and expression of MMP-8 in neutrophils upregulated by the AMPK pathway caused collagen to be destroyed, leading to lung consolidation [35]. They further found that hypoxia caused increased secretion of MMP-8, MMP-9, and NE in neutrophils, which in turn drives the destruction of major structural proteins of the lungs such as type I collagen, gelatin, and elastin, resulting in more severe tuberculosis tissue destruction [36]. Inflammatory bowel disease is a common clinical disease characterized by diffuse mucosal inflammation and neutrophil infiltration reaction. In the research and analysis of the correlation between the destruction of ECM and inflammatory bowel disease, it was found that the expression of MMP-2 and MMP-9 in the tissues of patients with inflammatory bowel disease was significantly higher than that of the control group. The rise of these MMPs caused the degradation and destruction of ECM protein and induced intestinal erosion and ulcer formation [37].

MMP-9, also known as gelatinase B, is an important member of the MMP family. It is currently believed that MMP-9/TIMP-1 is the main enzyme that regulates the degradation and synthesis of ECM. Studies have shown that the alveolar macrophages and neutrophils of smokers and COPD patients can release a large number of proteases and cytokines to increase the expression level and activity of MMP-9. MMP-9 not only causes ECM destruction but also degrades *α*1 antitrypsin and makes the balance of protease-antitrypsin imbalance, resulting in pulmonary matrix damage and lung dysplasia [38, 39]. In mouse colorectal cancer models, neutrophil-secreted MMP-9 activates latent TGF*β* in the ECM by degrading the ECM, increasing the level of TGF*β* in the tumor microenvironment, and thereby inhibiting antitumor T cell response [40]. Tissue inhibitor of metalloproteinase 1 (TIMP-1), a key regulator of MMP-9, blocks MMP-9 proteolytic activity. Related research found that pulmonary neutrophilia, hemorrhage, and vascular permeability were significantly higher in TIMP-1-deficient mice than in wild-type littermates. These results suggest that TIMP-1 may protect the alveolar-capillary barrier possibly by inhibiting proteolytic degradation of intercellular junctions or the alveolar basement membrane [41].

### 3.3. Cathepsins

Cathepsins (Cats) belong to lysosomal proteases, which maintain the homeostasis of the intracellular environment by hydrolyzing peptide chains to degrade proteins. According to the hydrolysis mechanism of the protease, the majority of Cat members are cysteine proteases, and a few are aspartate proteases and serine proteases. Based on its substrate specificity, Cat-A to Cat-Z have been reported to date. Cats mainly exist in lysosomes and play a role in proteolysis under physiological conditions. Under pathological conditions, cathepsins secreted into and out of cells increased, indicating that it is involved in a variety of physiological and pathological processes.

Cathepsin G (Cat-G) is a serine protease secreted by activated neutrophils, which has hydrolytic activity and plays a role in the inflammatory response. Atherosclerosis is a common chronic vascular inflammatory disease. Research has proved that Cat-G released by neutrophils can degrade elastin in the arterial wall to promote the formation of early atherosclerosis [42]. Protein evidence confirmed that the expression of Cat-G in atherosclerotic patients with aortic sclerosis was twice that of normal people. Excessive Cat-G mediates the cleavage of endothelial cadherin and fibronectin, resulting in the destruction of ECM and basement membrane structures, which causes endothelial dysfunction and vascular inflammation [43, 44]. Furthermore, Cat-G, which is stored in azurophilic granules of neutrophils as an inactive preform, displays various intracellular and extracellular functions related to tumorigenesis. Previous studies have shown that the related effects of Cat-G in tumors include ECM degradation [45], induction of immune cell chemotaxis [46], and increased tumor cell invasivity [47]. The present study confirmed that Cat-G markedly induced the formation of the E-cadherin/catenin complex on fibronectin, which increased the strength of cell-cell adhesion of MCF-7 human breast cancer cells, with important implications for tumor development and metastasis [48, 49].

### 3.4. Neutrophil Gelatinase-Associated Lipocalin

Neutrophil gelatinase-associated lipocalin (NGAL), also known as oncogene 24p3 or lipocalin 2, is a member of the lipocalin superfamily. NGAL is a 25-kilodalton protein of human neutrophils and can be covalently bound to gelatinases derived from neutrophils [50]. Studies in cultured cells and in murine models have revealed a pivotal role of NGAL in health and disease. Its small size, secreted nature, and relative stability have led to it being investigated as a diagnostic and prognostic biomarker in numerous diseases including inflammation and cancer [51, 52].

There is evidence that infiltrating neutrophils may amplify the immune response in cardiovascular injury [53, 54]. A recent study shows that NGAL produced by neutrophils can locally participate in cardiac remodeling and inflammation [52]. The increased expression of proinflammatory markers (MCP-1, OPN, and TNF-*α*) and collagen type I levels induced by NGAL in human cardiac fibroblasts highlight the pivotal role of NGAL in mediating cardiac damage. NGAL, as an inflammatory modulator of the innate immune system, plays a role in the progression of atherothrombotic disease by modulating the enzymatic activity of MMP-9 [55, 56]. Recent data demonstrate that NGAL may be a marker of atherosclerosis, which is supported by excessive expression of NGAL in atherosclerotic plaques in areas with high proteolytic activity [56, 57]. Studies have confirmed that the complex formation between NGAL and MMP-9 protects and prolongs the proteolytic activity of MMP-9, which increases the degradation of basement membranes and ECM, inducing plaque rupture and the risk of thrombus formation [56, 58]. Tumor-infiltrating neutrophils are an important source of NGAL. There have been reports that circulating blood levels of NGAL can be used as a potential marker for the detection and prediction of solid tumors and hematological malignancies [51]. Since MMP-9 is implicated in both early and late processes of tumor progression through the degradation of the extracellular matrix and basement membranes [59], whether NGAL and MMP-9·NGAL complexes can contribute to tumor progression is worthy of further investigation.

## 4. Regulation of ECM by Neutrophil Extracellular Traps

**Figure 1:**
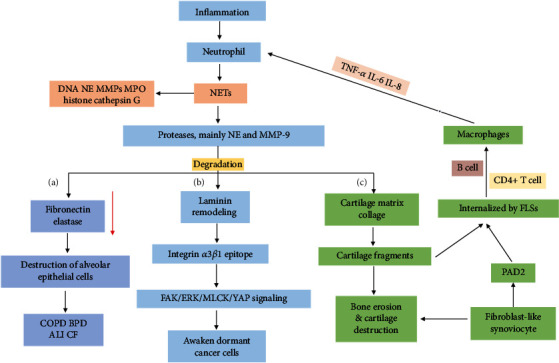
Regulation of ECM by neutrophil extracellular traps in diseases. NETs cleaved ECM in alveolar epithelial cells mainly via elastase and MMP-9, which destroyed the tight junctions between cells and increased tissue permeability, promoting the development of lung diseases. (b) Sustained inflammation induced the formation of neutrophil extracellular traps (NETs), and NET-associated proteases (NE, MMP-9) initiate awakening of dormant cancer cells by ECM remodeling. (c) Cytokines activate neutrophils to release elastase and MMP-9, which can directly degrade cartilage components in the synovium, promoting cartilage damage.

Activated neutrophils have recently been found to form neutrophil extracellular traps (NETs) that are involved in immune responses to pathogens [60]. NETs are composed of chromatin and granular proteins, including nuclear DNA, histones, MMP-9, myeloperoxidase (MPO), neutrophil elastase (NE), and cathepsin G. Since its discovery, the role of NETs in clearing pathogens has been widely confirmed. In recent years, new breakthroughs have been made in the study of NETs in inflammatory, autoimmune diseases and cancers (Figure 1).

Neutrophils have a stronger tendency to form NETs in patients with rheumatoid arthritis compared with healthy controls, and NE in NETs can directly degrade the cartilage matrix in the synovium, promoting cartilage destruction and synovial joint injury [61]. Experimental studies have shown that in a rat model of LPS-induced bronchopulmonary dysplasia (BPD), the massively formed NETs cleave fibronectin via NE and MMP-9 to further degrade ECM in the alveoli, thereby promoting the development of BPD [62]. Recently, the role of NETs in tumor progression has become a new focus of research. Research on the relationship between NETs and tumor progression is mostly based on neutrophil proteins (NE, MMP-9, etc.), which are also important components of NETs. It has been demonstrated that NE or MMP-9 in NETs can promote tumor growth and migration by degrading ECM. Therefore, it is speculated that NETs capture circulating tumor cells and expose them to a local microenvironment rich in active proteins, which is beneficial to the proliferation and metastasis of tumor cells. In addition, NETs itself can lead to increased recruitment of neutrophils to surrounding tumor cells, aggravating the inflammatory response, and promoting tumor recurrence and metastasis. Albrengues et al. reported that in an inflammatory environment, NETs bind to extracellular laminin and, due to the presence of laminin-degrading proteases like neutrophil elastase and MMP-9, induce the degradation of laminin [26]. This proteolytic processing leads to the exposure of a specific laminin epitope and triggering the activation of integrin and FAK/ERK/MLCK/YAP signaling, which results in the awakening and subsequent proliferation of dormant cancer cells at the metastatic sites. They further discovered that an inhibitory antibody against NETs remodeling laminin can prevent dormant cancer cells from being awakened.

## 5. Regulation of ECM by Neutrophil-Derived Exosomes

Exosomes are 40-120 nm membranous vesicles secreted by living cells, which carry bioactive factors such as lipids, proteins, and RNA components [63–65]. They are produced by different cells under physiological and pathological conditions. At present, it is believed that exosomes have many biological functions, not only mediating intercellular communication but also widely participating in different body reactions, including inflammation, immune signaling, cell proliferation, and differentiation [66, 67]. Neutrophil-derived exosomes are produced and released at rest or under the action of stimulating factors, which are closely related to multiple inflammatory diseases.

Neutrophil-derived exosomes can function independently of neutrophils, and they can also coordinate adaptive immune responses by affecting certain cell types. Researchers have found that exosomes play an indispensable role in lung disease. In addition to the discovery of exosomes in alveolar epithelial cells and macrophages, LPS-induced neutrophil-derived exosomes can increase the proliferation of airway smooth muscle cells and play a vital role in the progression of asthma and airway remodeling [68]. Recent studies have shown that exosomes can carry proteases with complete enzyme activity, which plays a key role in regulating the immune response [69, 70]. When stimulated by inflammation, CD63+/CD66b+ exosomes obtain surface-bound NE during neutrophil degranulation, which can be directed against the inhibitory effect of *α*1-antitrypsin (*α*1AT), thereby bypassing the pulmonary antiprotease barrier and promoting ECM destruction. Szul et al. have found that activated neutrophil-derived exosomes bind and degrade ECM in lung tissue via the integrin Mac-1 and NE, causing damage to the lung matrix and pulmonary dysplasia (Table 1), and promoting the occurrence of inflammatory diseases of the lungs, such as chronic obstructive pulmonary disease (COPD) [69]. A similar phenomenon has also been observed for another neutrophil-driven ECM remodeling disease (bronchopulmonary dysplasia (BPD)). Therefore, neutrophil-derived exosomes can promote ECM remodeling, aggravate the inflammatory responses, and destroy the body's normal immune defense system.

## 6. The Crucial Role of ECM and Neutrophil Interaction in Diseases

### 6.1. Infectious Diseases

Neutrophils are the primary effector cells in promoting destruction and preventing dissemination of fungal pathogens [77, 78]. Since mycotic infections occur within tissues, the response of extravasated neutrophils to fungal pathogens must take place in the context of the extracellular matrix. Recent studies demonstrated an important role for ECM in mediating the antifungal response of neutrophils [79, 80].

*Candida albicans* is the main pathogen causing fungemia and disseminated candidiasis [81]. It often grows in the form of a biofilm on medical biomaterials implanted in the body. A mature *Candida albicans* biofilm is a dense network system [82], composed of yeast cells, hyphae cells, and pseudohyphae cells wrapped in an extracellular matrix. ECM is crucial for maintaining the homeostasis of the cell environment, preventing dispersion of biofilms, and evading antifungal drugs [83]. Neutrophils are known to secrete toxic substance NETs to floating *Candida albicans*. Its components, such as DNA and granular proteins, can inhibit the growth and spread of *Candida albicans*. Cells in the biofilm are wrapped in the ECM produced by themselves, which is the main difference from planktonic cells. Studies by Johnson et al. suggest that biofilms can largely inhibit the release of NETs [80]. Impaired release of NETs leads to reduced bactericidal effects in vitro and in animal models of biofilm infection, which provides a novel mechanism for resisting neutrophil killing that is unique to the growth mode of biofilms. One of the main determinants of the biofilm driving this important immunosuppressive effect is the mannan-glucan complex in ECM [84]. By constructing a rat catheter model, they found that the knockout of the gene PMR1 encoding ECM mannan caused large amounts of NETs to be released, which indicated that the *Candida albicans* biofilm damaged the antifungal function of neutrophils through ECM-induced inhibition pathways, thereby resisting the attack of the body's immune system [80].

### 6.2. Chronic Inflammatory Diseases

Abnormal ECM expression and ECM fragments from tissue remodeling are reported to affect the behavior of immune cells in inflamed tissues, thereby promoting immune responses at the lesion site. Neutrophils are important effector cells that mediate inflammation responses, and their directed migration through the ECM is a complex multistep process. Previous studies have confirmed that during inflammation, the ECM protein lumican promotes the migration of neutrophils to the inflammatory site by binding to specific integrin receptors on the surface of neutrophils [85]. The study also found that a laminin *α*5 peptide can induce chemotactic migration of neutrophils and release MMP-9 [86].

It is worth noting that changes in the composition, content, and structural ordering of the ECM components are associated with the course of many chronic inflammatory diseases, especially several chronic lung diseases, such as COPD, idiopathic pulmonary fibrosis (IPF), and pulmonary hypertension (PAH) [87, 88]. The pathophysiological aspects of COPD are primarily caused by smoke exposure, including airflow obstruction and hyperexpansion. There is experimental evidence that structural and biomechanical changes in emphysema may arise from ECM remodeling within the alveolar walls [89]. In COPD, neutrophils are the most abundant leukocytes present in the bronchial walls and lumen, and they are also a major source of ECM-degrading enzymes. MMP-8 and MMP-9 are two MMPs implicated in the pathogenesis of tissue destruction in COPD [90]. Relevant literature shows that neutrophil-derived MMP-8 and MMP-9 cleave the collagen in the ECM to form the bioactive peptide of the proline-glycine-proline (PGP) sequence. PGP has structural homology with leukocyte interleukin 8, which activates CXCR1/2 receptors and causes neutrophil chemotaxis, thereby promoting the inflammatory cascade response of COPD [25, 34, 91]. Atherosclerosis (AS) is regarded as a chronic vascular inflammatory disease involving the immune system. In humans, atherosclerotic plaque instability is associated with the presence of intraplaque neutrophils. Neutrophils contribute to the progression of advanced AS by releasing granule proteins and forming NETs to degrade ECM [92]. During the development of AS lesions, neutrophils degrade large amounts of ECM, such as elastin, collagen, and fibrin, by releasing NE and MMPs (MMP-2, MMP-8, and MMP-9), leading to the occurrence of complications such as plaque rupture and thrombosis [75, 93]. In addition, numerous ROS released by neutrophils can increase the activity of MMP-8 and MMP-9, which enhance leukocyte aggregation and degradation of matrix components in the fibrous cap, leading to plaque instability and rupture [94].

### 6.3. Cancers

The progression of cancer involves not only the tumor cells themselves but also other participants, including immune cells recruited by cancer cells, the proinflammatory factors they release, and ECM [95]. ECM has various proteins and complex structures that can be used by tumor cells to create a tumorigenic environment. Matrix proteins including syndecan-1, collagen-IV, and laminin have been shown to be overexpressed in tumors and used as markers for tumor detection [96]. Neutrophils are important immune cells in the tumor inflammatory microenvironment. They rebuild the tumor microenvironment by releasing a large number of bioactive proteases and participate in the proliferation, invasion, metastasis, and angiogenesis of tumor cells.

ECM and neutrophils are considered as important parts of the tumor microenvironment, which significantly affects patients' therapeutic responses and overall outcomes [97, 98]. Tumor metastasis is a multistep dynamic process, and the destruction of the ECM structure around the tumor is a prerequisite for cancer cell metastasis [99, 100]. There is substantial evidence that neutrophil influx into developing tumors is associated with cancer progression and subsequent metastatic dissemination [97, 101, 102]. NE, a potent serine protease, is considered as a major inflammatory neutrophil product that directly modulates tumor cell behavior, tumor-host interactions, and key initial steps in the metastatic cascade. Reports have demonstrated that excess NE released by neutrophils in lung cancer promotes the invasion and metastasis of cancer cells by destroying the intrinsic tissue barrier composed of the basement membrane and other ECM components (including elastin, collagen, and proteoglycan) [71, 72]. In addition, neutrophil-derived MMP-9 is viewed as a major player in the vascularisation of tumors [103, 104]. TGF*β* in the tumor microenvironment can induce the polarization of N2 tumor-associated neutrophils and produce a large amount of MMP-9 and VEGF. MMP-9 induces the cleavage of laminin and collagen within the basement membrane and ECM, which promotes the movement of endothelial cells and pericytes and ECM remodeling that is critical for tumor angiogenesis [104, 105]. Recently, a new study showed that NETs formed by neutrophils in the process of continuous lung inflammation awaken dormant breast and prostate cancer cells, leading to the formation of metastatic tumors in the lungs [26]. ECM is associated with awakening dormant cancer cells and cancer metastasis. In this study, NE and MMP-9 in NETs sequentially cleaved laminin in tissues, thereby exposing a specific integrin *α*3*β*1-activated epitope to induce proliferation and metastasis of dormant cancer cells.

## 7. Conclusions

Cell-matrix interactions are essential for proper cell and tissue functionality. Recent studies have shown that ECM is inseparable from the immune system and immune response. As a potential organizer of immune cell compartments, the basic structure of ECM and its physiological and pathological functions play a vital role in the establishment and maintenance of immune homeostasis. The past 5 years have brought major advances in defining the role of neutrophils in the degradation and remodeling of ECM, which has exciting implications for a better comprehension of the interactions between ECM and neutrophils. However, gaps remain in understanding the regulatory role of ECM in determining neutrophil function. There are increasing studies indicating that the regulatory role of neutrophils in determining ECM remodeling is of considerable importance in the occurrence and development of various diseases. Neutrophils, with their large number of potent proteinases, play vital roles in the extensive remodeling of ECM macromolecule networks. In turn, ECM involvement in regulating neutrophil function in tissues makes accounting for both the presence of ECM and the interplay between immune cells and their extracellular matrices an important and outstanding mechanistic aspect of inflammation. For example, ECM can remodel the inflammatory microenvironment and tumor immunity by modulating the migration of neutrophils. Although we have a certain understanding of the effects of the abundant ECM protein and its biological function on neutrophils, there are still many important questions to be answered in this field. In addition, over the last years a plethora of ECM molecules have emerged as valuable targets for disease prognosis and treatment. A deeper understanding of the multiple interactions between ECM and neutrophils and their effects on cell signaling are fundamental. Hence, more studies are required to explore and understand the neutrophil-matrix interactions that occur during tissue homeostasis and infectious diseases, inflammatory diseases, and malignancy. It will be important to unravel the underlying mechanism of the interplay between immune cells and their extracellular matrices, which may be further exploited in the treatment of patients with inflammatory or infectious diseases in the near future.

## Figures and Tables

**Table 1:** Effects of neutrophil- released factors on extracellular matrix function.

Neutrophil-released factors	Effects on ECM	Diseases	References
NE	Degradation of ECM components (elastin, collagen, fibronectin, and proteoglycan)	COPD, IBD	[69, 33]
	Destroys the tight junctions between cells and increases tissue permeability	ALI, ARDS, lung cancer, etc.	[32, 71, 72]
	Protease-antiprotease imbalance		

MMPs	Upregulation of MMP-8 and MMP-9 leads to the degradation of lung structural proteins (collagen and elastin)	TB	[35, 36]
	MMP-9·NGAL complexes protect and prolong the proteolytic activity of MMP-9	OA	[73]
	Upregulation of MMP-1, MMP-8, and MMP-9	Tumor metastasis	[74, 75]
	Degrade the fibrous caps and collagen, which promote plaque instability and rupture	Atherosclerosis	[76]

Cathepsin G	Cleavage of endothelial cadherin and fibronectin		[43]
	Activation of MMPs, which increases TNF-*α* and IL-8	Atherosclerosis	[44]
	Induce E-cadherin/catenin complex formation and improve the cell adhesion strength of E-cadherin-mediated MCF-7 cells	Breast cancer	[48]

NETs	Bind to the extracellular laminin, trigger integrin and FAK/ERK/MLCK/YAP signaling, and awaken dormant cancer cells	Lung cancer	[26]
	NE and MMP-9 cleave ECM proteins	BPD	[62]
	NE degrades cartilage matrix	RA	[61]

Exosomes	Degrade ECM by binding to NE and integrin Mac-1	COPD	[69]
	Resist the inhibitory effect of *α*1-antitrypsin	BPD Asthma	[68]

*Note*. ECM: extracellular matrix; BM: basement membrane; NE: neutrophil elastase; MMPs: matrix metalloproteinases; NETs: neutrophil extracellular traps; TNF-*α*: tumor necrosis factor alpha; IL-8: interleukin-8; MMP-9: matrix metalloproteinase-9; NGAL: neutrophil gelatinase-associated lipocalin; COPD: chronic obstructive pulmonary disease; IBD: inflammatory bowel disease; ALI: acute lung injury; ARDS: acute respiratory distress syndrome; BPD: bronchopulmonary dysplasia; RA: rheumatoid arthritis; OA: osteoarthritis; TB: tuberculosis.
